# Acupuncture for blunt chest trauma: A protocol for a double-blind randomized control trial

**DOI:** 10.1097/MD.0000000000025667

**Published:** 2021-05-07

**Authors:** Pei-Yu Kao, Eyal Ben-Arie, Ting-Yu Lu, Wen-Chao Ho, Yu-Chen Lee, Yu-Sen Lin, Chien-Kuang Chen, Jian-Xun Chen, Tzu-Min Huang, Fang-Pey Chen

**Affiliations:** a Division of Thoracic Surgery, Department of Surgery, China Medical University Hospital, Taichung; b Institute of Traditional Medicine, School of Medicine, National Yang-Ming University, Taipei; c Graduate Institute of Acupuncture Science, China Medical University; d Department of Public Health, China Medical University; e Department of Acupuncture, China Medical University Hospital, Taichung; f Center for Traditional Medicine, Taipei Veterans General Hospital, Taipei, Taiwan.

**Keywords:** acupuncture, blunt chest trauma, press tack needle, rib fracture

## Abstract

**Introduction:**

Blunt chest trauma (BCT) accounts for up to 65% of polytrauma patients. In patients with 0 to 2 rib fractures, treatment interventions are typically limited to oral analgesics and breathing exercises. Patients suffering from BCT experience symptoms of severe pain, poor sleep, and inability to perform simple daily life activities for an extended period of time thereafter. In this trial, we aim to investigate the efficacy of acupuncture as a functional and reliable treatment option for blunt chest trauma patients.

**Methods:**

The study is designed as a double-blind randomized control trial. We will include 72 patients divided into 2 groups; the acupuncture group (Acu) and placebo group (Con). The acupuncture group will receive true acupuncture using a uniquely designed press tack needle. The control group will receive placebo acupuncture treatment through the use of a similarly designed press tack needle without the needle element. The acupoints selected for both groups are GB 34, GB 36, LI 4, LU 7, ST 36, and TH 5. Both groups will receive 1 treatment only following the initial visit to the medical facility and upon diagnosis of BCT. Patient outcome measurements include: Numerical Rating Scale, Face Rating Scale, respiratory function flowmeter, Verran Snyder-Halpern sleep scale, and the total amount of allopathic medication used. Follow-up time will be scheduled at 4 days, 2 weeks, and lastly 3 months.

**Expected outcome:**

The results of this study can potentially provide a simple and cost-effective analgesic solution to blunt chest trauma patients. This novel study design can serve as supporting evidence for future double-blind studies within the field of acupuncture.

**Other information:**

The study will be conducted in the thoracic surgical department and acupuncture department in China Medical University Hospital, Taichung, Taiwan. The study will be conducted on blunt chest trauma patients and is anticipated to have minimum risk of adverse events. Enrollment of the patients and data collection will start from March 2020. Study completion time is expected in March 2022.

**Protocol Registration:**

(CMUH109-REC1-002), (NCT04318496).

## 1 Introduction

The rib cage and associated anatomical structures are responsible for protecting the vital organs located within the chest cavity.^[1]^ The occurrence of blunt chest trauma (BCT) typically involves multiple levels of injury including heart rupture, aortic dissection, lung contusion, rib fracture, or simple soft tissue injury. In the case of preserved organ function, it is the bony structures and soft tissues which are most commonly affected by BCT injury.^[1]^ According to previous literature, BCT patients can be divided into 3 levels based on the severity of injury as identified by chest plain film findings. Level 1 includes patients without rib fractures; level 2 includes patients with 1 or 2 rib fractures; and level 3 includes patients with more than 2 rib fractures.^[2]^ Based on retrospective studies, level 3 patients experience increased frequency of hospitalization, thoracotomies, and a higher mortality rate. Levels 1 and 2 patients are commonly treated as out-patients, but still suffer greatly from chest pain and tend to experience breathing difficulties.^[2,3]^ The pain associated with the BCT injury may in turn lead to the inability have sufficient sleep, with the resultant lack of rest impeding the recovery process.^[4]^ The most commonly used conventional treatments are analgesics.^[1,5]^ Moreover, while nerve block surgical intervention is also practiced as a treatment intervention, it is commonly reserved for level 3 patients experiencing severe pain and/or thoracic deformity.^[1,5]^ While analgesic medication can relieve pain temporarily, most patients still suffer prolonged nociceptive hypersensitivity and usually have difficulties in performing daily life activities for sometime after. Furthermore, elderly and renal insufficiency patients are at a higher risk of experiencing harmful effects from long term use of analgesics and muscle relaxants.^[6]^

For thousands of years, acupuncture has been used in the treatment of all kinds of traumatic injuries. Moreover, in recent decades, stimulation of acupuncture points for the treatment of visceral pain, post-operative pain, cancer pain, and chronic pain is well established and has been widely investigated in a number of clinical trials.^[7–9]^ Accordingly, in order to maintain an unbiased approach, the acupuncture intervention of this particular study will be compared to a placebo intervention. Our study implements a novel design that maintains clinical efficacy, through the use of press tack needles and similar placebos that look identical, hereby ensuring blindness of both the patient and the acupuncturist.^[10]^

With a goal to improve the analgesic effect and life quality in patients suffering from BCT, we designed a double-blind study comparing press tack acupuncture to press tack placebo interventions. In the trial, we aim to investigate the efficacy of press tack acupuncture based on traditional acupuncture concepts and point locations. Furthermore, we hypothesize that press tack acupuncture will have a superior analgesic effect when compared to press tack placebos.

## 2 Methods

### 2.1 Design and setting

The study is designed as a single-center randomized control, parallel-arm, double-blinded trial. It will take place in the out-patient clinic of China Medical University Hospital. The study will be conducted from March 2020 to March 2022. The study was approved by the institutional review board of China Medical University Hospital (CMUH109-REC1-002), and was registered on clinicaltirl.gov (NCT04318496).

The main goal of the study is to reduce pain and improve the respiratory function and sleep quality of patients after BCT. The participants will be divided to 2 groups; acupuncture group (Acu) who will receive the true press tack needles, and a placebo group (Con) who will receive press tack placebos on the same acupoints without the needle element. Patient outcome measurements include: Numerical Rating Scale (NRS), Face Rating Scale (FRS), respiratory function flowmeter Verran Snyder-Halpern sleep scale (VSH), and the total amount of allopathic medication used.

### 2.2 Participants

We will include a total of 72 patients who are suffering from BCT. After the patient's accident, such as falls, kicks, fights, or traffic accidents, they will be sent to the emergency room and visit a chest surgical out-patient clinic for treatment and follow-up. Those who meet the inclusion criteria will be informed about the study and can freely choose to participate. They will sign the informed consent and will be randomized accordingly, with commencement of the study occurring immediately. The patients are allowed to drop out from the study at any time point of the study.

### 2.3 Inclusion criteria

- Age 20 to 80 years old.
- Patients who have chest trauma as described by themselves or based on medical chart records within 1 week of injury.
- Injury Severity Score (ISS) is less than 18 points.
- Body mass index < 30.

### 2.4 Exclusion criteria

- Sternal fracture.
- ISS is equal to or more than 18 points.
- History of intercostal nerve injury.
- History of cardiovascular disease.
- History of chronic lung disease.
- Significant lung mass or chest deformity noted in the chest plain film.

### 2.5 Informed consent

From the informed consent discussion and form, the intervention, risks, benefits, and patient's rights in the study will be explained in a simple language that patients can clearly understand (by an hospital doctor). The informed consent will be signed by the patient or by a legal guardian. In the circumstance that they wish to withdraw from the study, our strategy is to use the available data for the final analyses and accommodate the patients’ wishes accordingly.

### 2.6 Randomization and allocation concealment

The participants will be randomized into 2 groups with a 1:1 ratio produced by the SPSS program software, version 22. A computer-based simple random sampling with a 1:1 ratio without stratification will be used. A number (1–72) will be affiliated to each patient at the time of their first visit to the out-patient clinic and after completing the informed consent. The number, patient name, and date of birth will be written down on a non-transparent envelope by a study nurse 1 (researcher EB-A). Inside the envelopes, there will be 14 separate sterile press tack needles or identical looking press tack placebos. The envelopes in both groups will appear identical. Study nurse 1 will write down the number, patient name, and intervention in an electronic chart and create a file for each patient.

### 2.7 Blinding

Study nurse 1 will be responsible for randomization and prepare the needles in the envelopes. Outside the envelopes, there will be a number belonging to 1 patient, and inside the envelopes, there will 14 separate sterile needles which can be either true press tack needles or press tack placebos. The envelopes and the pouch of needles will look the same. The acupuncturist will be asked not to check the needles when they insert them. At the time of acupuncture, a nurse will accompany the acupuncturist and will verify that he/she is not examining the needles closely. Study nurse 2 will perform the outcome measurement tests. The acupuncturist, patients, and study nurse 2 will be blinded to patient allocation.

### 2.8 Procedure/interventions

**Figure 1:**
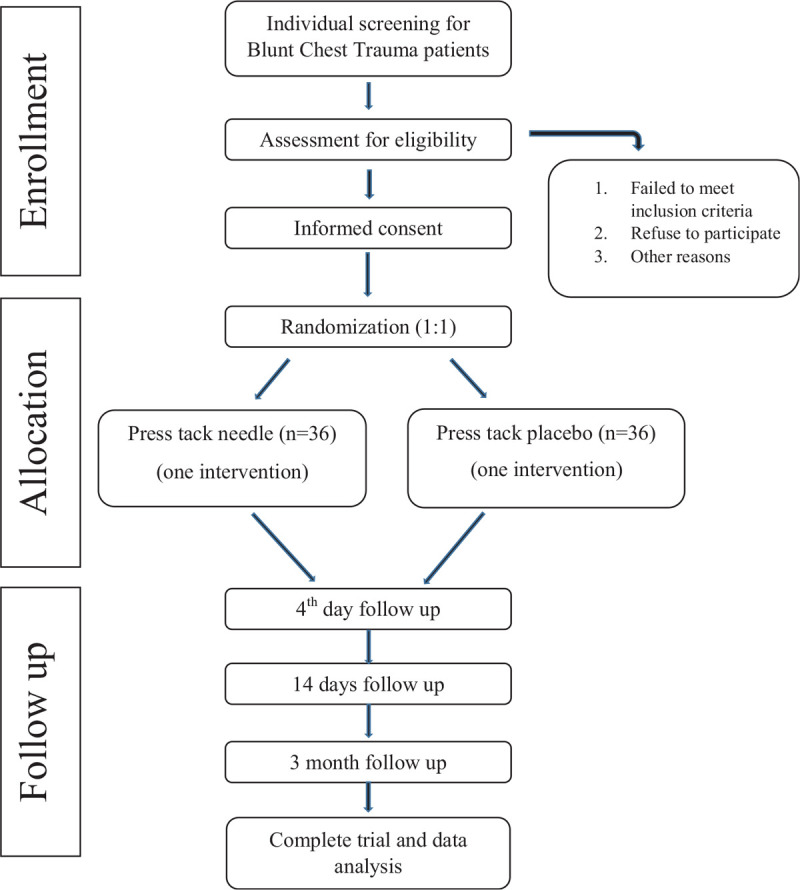
The figure describes the trial processes at a descending order.

The participants will be divided into 2 groups. One group will receive press tack needle acupuncture (Acu); the other group will receive press tack needle placebos without the needle element (Con). Both groups will receive 2 individual treatments on the same set of acupoints. The needle retention time will be 4 days. Needle withdrawal will be done by the acupuncturist or acupuncture nurses who have trained in needle withdrawal techniques in the out-patient clinic (Fig. 1).

### 2.9 Press tack needle introduction

In the protocol, the press tack needles (PYONEX*Φ*0.20 mm × 0.6 mm made by Seirin Corporation) have a diameter of 0.2 mm and a length of 0.6 mm. Each needle is packed separately in a sterilized pouch. As for the placebo, the individual press tack and pack are identical to the press needles, except the needle element has been removed. Both interventions will be performed by a qualified acupuncturist and a medical doctor with at least 1 year of experience in acupuncture practice.

### 2.10 Acupuncture group (Acu)

The patient will lay down in a supine position on the treatment bed and allowed an adaptive relaxation period of 5 minutes. The acupuncturist will start to sterilize the specific body surface areas over the acupoints with a 70% alcohol pad. Accordingly, the bilateral acupoints of GB 36 (Waiqiu), GB 34 (YangLingQuan), ST 36 (Zusanli), LI 4 (HeGu), LU 7 (LieQue), TH 5 (Waiguan) were chosen for both groups. Each patient will receive 12 press tack needles in total. The needle's depth of insertion will be 0.6 mm. The acupuncturist will manually press the press tack needles over the above-mentioned acupoints without any needle manipulation or “De Qi” sensation. The participants will lay on the treatment bed for the duration of 30 minutes. After the aforementioned process, the vital signs, pain levels, and flowmeter will be recorded again to represent the immediate effect after acupuncture. Finally, the acupuncturist will educate the patients about the total number of press needles and the allotted time of retention. The press tack needle retention time will total 4 consecutive days.

### 2.11 Placebo group (Con)

**Figure 2:**
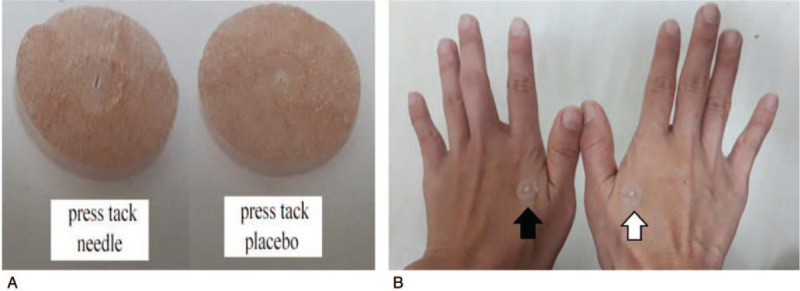
(A) Press tack needles have an adhesive surface with a needle diameter of 0.2 mm and a length of 0.6 mm. Press tack placebos have an identical adhesive surface without a needle (Pyonex; Seirin Corporation, Shizuoka, Japan). (B) The outer surface of both press tack needles and press tack placebos looks identical.

**Figure 3:**
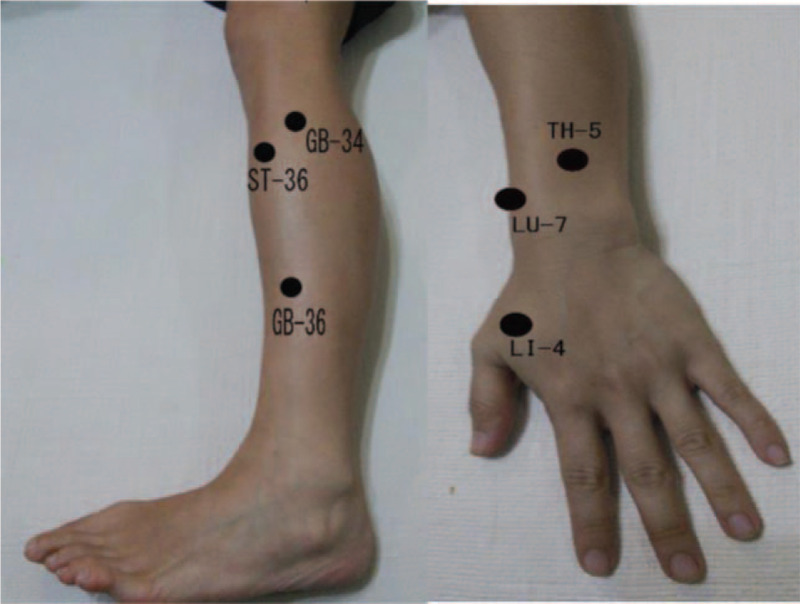
Acupoints used in the trial for both groups: GB 36 (Waiqiu), GB 34 (YangLingQuan), ST 36 (Zusanli), LI 4 (HeGu), LU 7 (LieQue), TH 5 (Waiguan).

The participants in the placebo group will experience the same treatment course including the selection of acupoints and associated outcome measurement recording. However, the placebo group participants will receive press tack placebos (Pyonex; Seirin Corporation, Shizuoka, Japan). The press tack stickers on the skin surface are identical but without any needle penetrating the skin. The placebo sticker retention time will be the same as in the acupuncture group, totaling 4 consecutive days (Figs. 2 and 3).

### 2.12 Drug treatment

Both groups of participates will also receive the prescribed analgeasic medication and/or muscle relaxants. The frequency of the prescribed medicine is 3 to 4 times a day along with meals and before sleep for 7 days. The purpose of the medication is for pain relief, therefore, the participants can discontinue the medication at will.

### 2.13 Outcome measures

The main outcome measurement will be the pain levels of both rest and mobile status for the BCT patients (the mobile status includes cough, deep breath, and turn over on the bed). The patient will measure their pain according to 3 subjective pain scales, namely the NRS, and FRS before treatment, 30 minutes after treatment and 4 days after the press tack needles/placebo intervention. Furthermore, a follow-up will assess the NRS pain scale telephonically at 14 days and 3 months after the initial intervention.

Secondary outcome measurements will include the improvement of respiratory function, sleep quality, and amount of medication used (specifically analgesics and muscle relaxants). We used the portable triflow flowmeter and VSH sleep scale as objective tools for the aforementioned purposes. The patients will be educated on the use of the flowmeter by the affiliated thoracic surgeon. Patients will be allowed as much time as needed to practice the use of the flowmeter before the first measurement is taken. When the patient is ready, the study personnel will commence the measurements of 3 attempts, and will record the best 2 attempts. The triflow flowmeter measures the amount of air the patient can exhale; it has 3 chambers with their own balls that require air pressures from 600 to 1200 cm^3^ per second to elevate. 1200 cm^3^ is the highest score and indicates good exhalation power. The above process will be done before intervention, 30 minutes after intervention and 4 days after the intervention. The patient will also answer the VSH sleep scale in Mandarin to evaluate the sleep quality of the night before the clinic visit. They will complete the questionnaires accordingly at every clinic visit, for a total of 2 times (on day 1 and day 4). Finally, on the 4th day after the initial intervention, participants will bring back the prescribed medication, including analgesics and/or muscle relaxants that were left unused, to be measured for the amount of medication used.

### 2.14 Follow-up

**Figure 4:**
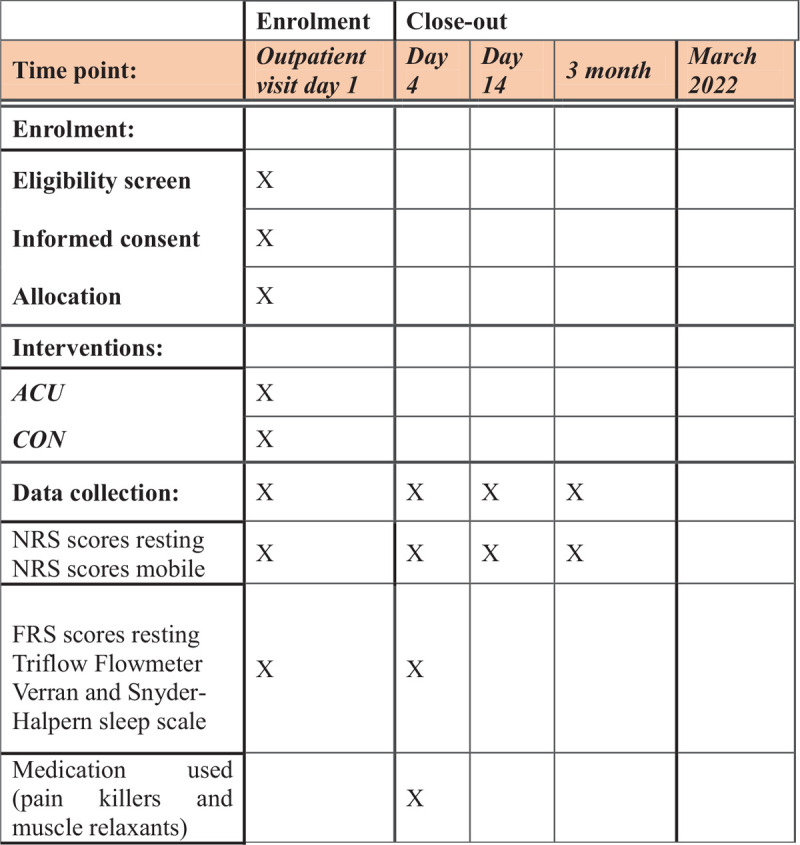
The figure describes the trial schedule from patients enrollment to trial close-out.

After the initial visit to the chest surgical out-patient clinic, the participates in both the Acu and Con groups will be expected to return 4 days after acupuncture/placebo treatments for needles/placebos withdrawal along with chest plain film, NRS, FRS scores, flowmeter, respiratory function, VSH sleep scale assessments, and amount of medication used. On the 14th day and at 3 months after the initial intervention, a study personal will arrange a telephonic consultation to enquire about the pain scale (NRS) and any adverse effect related to the acupuncture treatment and the chest trauma (Fig. 4).

### 2.15 Adverse events

Before the enrollment of the study, the participants or the patient's legal guardian will be informed about acupuncture adverse events including bleeding, hematoma, infections, alcohol allergy, pneumothorax, etc. In any case of acupuncture related serious adverse events occurring, these will be reported immediately to the primary investigator and the institutional review board. We will collect all the adverse event details such as: treatment site, acupuncture points selected, patient recovery outcome, number of events, etc. Acupuncture will be stopped and treatment groups will be un-blinded. We will also provide suitable treatment, including hospitalization for the patients.

### 2.16 Sample size calculation

In a previous similar study,^[11]^ the pain scale reduced significantly as recorded immediately after acupuncture treatment. We utilized the result of this study to calculate sample size as generated by G∗power version 3.1.9.2 software. Accordingly, in the condition of 95% power being calculated with an effect size of 1.124365. The patient number in each group should be a minimum of 18 participants. However, we also considered that a previous study included 29 patients in each group with a total of 58 patients. With respect to a dropout rate of 20%, we decided to include 36 patients in each group, for a total of 72 patients.

### 2.17 Quality control/data management

To maintain the integrity of the study, all the recorded data and documents will be recorded manually as a personal file first and restored in both paper and electronic form. All the documents will be stored in a secure location in the chest surgical department of China Medical University Hospital, and will be destroyed after 5 years of trial completion. Access to patient data will be restricted to study medical personals. We will also arrange regular meetings to gather the entire study personnel, including the primary investigator, the acupuncturist, study nurses, and chest surgeons, in order ensure the integrity of the study protocol and due process. Any modification in the study protocol will be done upon agreement with the China Medical University Hospital institutional review board.

### 2.18 Dissemination

After the completion of the study, we plan to publish the results and the study data in a medical journal.

### 2.19 Statistical analysis

Statistical analyses will be performed using SPSS software version 22.0 (SPSS Inc, Chicago, IL). The demographic data between Acu and Con groups including gender, age, and clinical characteristics will be compared using the Student's *t* test and Chi-squared test. Between-group values will be measured by the paired *t* test. For the multiple pain scale and sleeping quality measurements, a one-way ANOVA will be used according to the previous similar study.^[11]^ We will also implement the advanced repeat measurement analysis in the study. Data will be presented as mean ± standard deviation. A *P*-value of less than .05 will be considered statistically significant.

Subgroup analysis will be done based on the following: age, sex, number of treatments complete, number of hours of needle retention, number of rib fractures, pulmonary complications, hospitalization, number of days from patient's injury to the first treatment, Triflow score, medication use, body mass index, and ISS. In cases of small sample size, the Mann–Whitney *U* and the Wilcoxon sign rank test will be implemented accordingly. Missing data will be analyzed using the pairwise deletion method.

## 3 Discussion

The nociceptive response is a reflexive bodily response of the body aimed at providing protective mechanisms. In short, pain creates awareness of pathological conditions resultant from injury or harm, and may vary in intensity, quality, duration, and referral. Accordingly, pain is categorized according to 4 distinct types: nociceptive pain, inflammatory pain, neuropathic pain, and functional pain.^[12]^ In the case of BCT, nociceptive signals are transmitted to the brain following peripheral tissue damage. In terms of the 1 to 10 NRS pain scale, the patient's pain levels on the first recorded day of injury can reach up to 9 to 10 points, whereafter the pain sensation gradually decreases and is subsequently recorded at reduced levels of 2 to 3 points after 15 days.^[13]^ However, the long term effects of pain resultant from physical injury usually display concurrent overlapping conditions related to the physical, psychological, and even socio-economic function ability of patients, and can last up 12 months after initial injury.^[14]^

Acupuncture treatment is well known for its analgesic potential, with proven efficacy particularly in chronic pain conditions. Furthermore, the therapeutic benefits of this treatment are extended to complicated conditions of maladaptive pain, such as myofascial pain, tension headache, irritable bowel syndrome, etc.^[15–19]^ In terms of acute pain, acupuncture has displayed promising analgesic effects in a wide array of diseases, with particular emphasis on emergency medicine.^[20–22]^ Furthermore, acupuncture has displayed tendencies of increased efficacy in the treatment of bone fractures as shown in elders in the acute stage of hip fracture. In addition, patients who received acupuncture treatment in the ambulance en route to hospital, reported reduced pain sensations, less anxiety, and lower heart rates. They also stated a higher overall satisfaction regarding the emergency ride to the hospital.^[22]^

The analgesic effects of acupuncture have been further biochemically described via research obtained from various animal studies. The underlying molecular mechanisms of acupuncture analgesia are commonly associated with an increase in adenosine and an upregulation of the A1 receptors that are largely associated with anti-nociceptive effects.^[23]^ Other possible explanations of acupuncture analgesia include the evident reduction in common inflammatory mediators such as substance P, IL-1β, IL-8, IL-10, and TNF-α, all of which are over-expressed in nociceptive hypersensitive states, specifically inflammatory pain, and functional pain conditions.^[24]^

Current literature contains limited information with regards to the use of acupuncture treatment in cases of BCT. Accordingly, only 1 randomized control trial and 1 case report about the benefit of acupuncture for the blunt chest injury was discovered.^[11,25]^ In the randomized control trial, Ho et al focused on the patients with 2 or more rib fractures requiring hospital admission (level 3). These patients were usually well monitored and treated with adequate analgesic mediations during their hospital stay. When patients classified according to level 1 or level 2, without rib fractures or with less than 2 fractures, respectively, are treated in the out-patient clinic, the pain control measures are limited, especially when intravenous (IV) medications are not available.

To this end, acupuncture provides suitable, reliable, and effective forms of relief for these patients. The efficacy of Chinese Medicine in the treatment of various conditions has been proven over thousands of years.^[26]^ Doctors discovered that the injuries of internal organs or the torso could be treated via the stimulation of the distal skin surface, such as the limbs, ears, or skull. They drew lines to connect specific points, and developed a map of the 14 meridians, each of them is related to a specific organ system.^[27]^ In the present study, we utilize this concept through the use of distal point stimulation of 4 limbs to ease the chest pain associated with traumatic incidences without directly applying needles at the site of injury.

With regards to the manipulation of acupuncture needles for increased therapeutic benefit, a multitude of options are available, including manual acupuncture, electro-acupuncture, press tack needle, laser acupuncture, etc.^[28]^ For this protocol, we had chosen the press tack needles and press tack placebo due to their simplicity and similarity which allows for effective blinding. The press tack needles look identical to the press tack placebo, whereby one can only see a difference at the time of needle insertion and withdrawal upon extremely close inspection of the needles, and thus they can serve as an ideal device to generate the “blindness” effect to both the patients and the acupuncturist.^[10]^ The “De Qi” phenomena, which is usually achieved through manipulation of the acupuncture needles via rotation is considered by some as a major part of the acupuncture effect, and might be lost in the application of press tack needles, thus being regarded as the press tack needles’ biggest disadvantage.^[29,30]^ However, as we want to apply the use of acupuncture in a conventional allopathic, we believe that the press tack needles can provide a simple to use and safe treatment option for all modalities of medical care. In this study protocol, we decided to implement the following acupoints: GB 34 (YangLingQuan), GB 36 (Waiqiu), LI 4 (HeGu), LU 7 (LieQue), ST 36 (Zusanli), TH 5 (Waiguan), as a result of similar theoretical affiliations. Specifically, the concept associated with these specific points is directed at the fact that most chest trauma occurs on the frontal and lateral areas of the chest. Accordingly, Chinese medicine theory describes the frontal area of the chest as being controlled by the yang ming layer (stomach and large intestine meridians), to which end the acupoints: LI 4 (HeGu) and ST 36 (Zusanli) belong to that layer and can potentially generate an analgesic effect within the specified area. The lateral side of the chest is purportedly controlled by the shao yang layer (gall bladder and triple warmer meridians), whereby the acupoints GB 34 (YangLingQuan), GB 36 (Waiqiu), and TH 5 (Waiguan) can potentially improve Qi and blood flow within this specific region and alleviate pain.^[29,31]^ The addition of the acupoint LU 7 (LieQue) was designed to treat the lung organ and improve lung functions.^[32]^ The acupoints ST 36 (Zusanli) and LI 4 (HeGu) were found to decrease incisional pain and improve post-operative rest in post abdominal surgery patients.^[33]^ The acupoints LU 7 (LieQue), LI 4 (HeGu), ST 36 (Zusanli), TH 5 (Waiguan) also showed a general analgesic benefit and increased post-operative recovery.^[34]^

### 3.1 Limitations

The study provides an effective intervention but is limited to a shallow acupuncture needling depth of the superficial subcutaneous layers and cannot be associated with the benefits caused by the deeper stimulations commonly associated with most acupuncture interventions. Furthermore, the point selection in this study only concentrates on anterior and lateral BCT locations. Lastly, the follow-up method of telephone calls at 14 days and after 3 months can prove problematic, and a high loss to follow-up is anticipated.

In this study, we offer a simple, low-cost form of acupuncture that, if it will prove superior analgesic effects in comparison to the placebo, can provide a safe and reasonable treatment alternative to BCT patients.

## Acknowledgments

We would like to acknowledge the Seirin Corporation for providing the acupuncture and placebo press tack needles. We would also like to acknowledge Dr Bernice Lottering for her assistance in the editing of this paper.

## Author contributions

**Analysis and interpretation of the data:** Ho Wen-Chao, Eyal Ben-Arie, Kao Pei-Yu.

**Conceptualization:** Pei-Yu Kao, Eyal Ben-Arie, Yu-Chen Lee, Fang-Pey Chen.

**Data curation:** Pei-Yu Kao, Wen-Chao Ho, Fang-Pey Chen.

**Design:** Eyal Ben-Arie, Kao Pei-Yu, Lee Yu Chen, Fang-Pey Chen.

**Formal analysis:** Wen-Chao Ho.

**Funding acquisition:** Kao Pei-Yu, Fang-Pey Chen.

**Investigation:** Pei-Yu Kao, Ting-Yu Lu, Yu-Sen Lin, Chien-Kuang Chen, Jian-Xun Chen, Tzu-Min Huang.

**Methodology:** Pei-Yu Kao, Eyal Ben-Arie, Wen-Chao Ho, Yu-Chen Lee, Fang-Pey Chen.

**Patients enrollment:** Kao Pei-Yu, Ting-Yu Lu, Yu-Sen Lin, Chien-Kuang Chen, Jian-Xun Chen, Tzu-Min Huang.

**Project administration:** Pei-Yu Kao, Eyal Ben-Arie, Yu-Chen Lee, Fang-Pey Chen.

**Resources:** Pei-Yu Kao, Yu-Chen Lee, Fang-Pey Chen.

**Software:** Eyal Ben-Arie, Kao Pei-Yu, Ho Wen-Chao.

**Supervision:** Pei-Yu Kao, Yu-Chen Lee, Fang-Pey Chen.

**Validation:** Wen-Chao Ho, Kao Pei-Yu.

**Visualization:** Pei-Yu Kao

**Writing – original draft:** Pei-Yu Kao, Eyal Ben-Arie.

**Writing – review & editing:** Pei-Yu Kao, Eyal Ben-Arie, Yu-Chen Lee, Fang-Pey Chen.
